# Science in the fight to uphold the rights of children

**DOI:** 10.1371/journal.pbio.3000010

**Published:** 2018-09-18

**Authors:** Arthur L. Caplan, Peter J. Hotez

**Affiliations:** 1 Division of Medical Ethics, New York University School of Medicine, New York, New York, United States of America; 2 Texas Children’s Hospital Center for Vaccine Development, Departments of Pediatrics and Molecular Virology and Microbiology, National School of Tropical Medicine, Baylor College of Medicine, Houston, Texas, United States of America; 3 Department of Biology, Baylor University, Waco, Texas, United States of America; 4 James A Baker III Institute of Public Policy, Rice University, Houston, Texas, United States of America; 5 Scowcroft Institute of International Affairs, Bush School of Government and Public Policy, Texas A&M University, College Station, Texas, United States of America

## Abstract

The United States is the only major nation to not yet have ratified the United Nations Convention on the Rights of the Child (UNCRC). Recently, there has been an erosion of the rights of children across America, Europe, and elsewhere, but through science, we may have an opportunity to counter some of this alarming trend. In the area of vaccines, the scientific community can raise its voice on the dangers that nonmedical exemptions and delays pose to children at risk for measles, influenza, and other childhood illnesses. Poverty places infants and children at high risk for illness and homelessness. Gun violence and gun-related accidents are killing on average four American children daily, and climate change is promoting global pediatric malnutrition. Increasing international, federal, and state support to seek innovative solutions to these and related issues is a moral imperative.

## Abstract 2

In this Perspective article, Peter Hotez and Arthur Caplan argue that scientists can have important and new roles in limiting recent declines in the rights of children across America (and in some European countries), especially in the areas of child poverty, low vaccination rates, gun violence, and disease and malnutrition due to climate change.

In 1989, the United Nations (UN) General Assembly adopted the UN Convention on the Rights of the Child (UNCRC), a treaty recognizing human rights for the world’s children [1]. Working under the Office of the UN High Commissioner for Human Rights (OHCHR), some of the major articles of the UNCRC include those specifying that every child has an “inherent right to life” and others ensuring “the survival and development of the child” [1]. There are also specified articles for combating disease, diminishing child mortality, “abolishing traditional practices prejudicial to the health of children,” and establishment of a “framework of primary health care” [1]. As shown in Fig 1, almost every nation has signed and ratified the UNCRC, with the important exemption being the United States. Thus, while the US had an important role in creating the language for the Convention, so far, the US has failed to ratify it [2].

**Fig 1:**
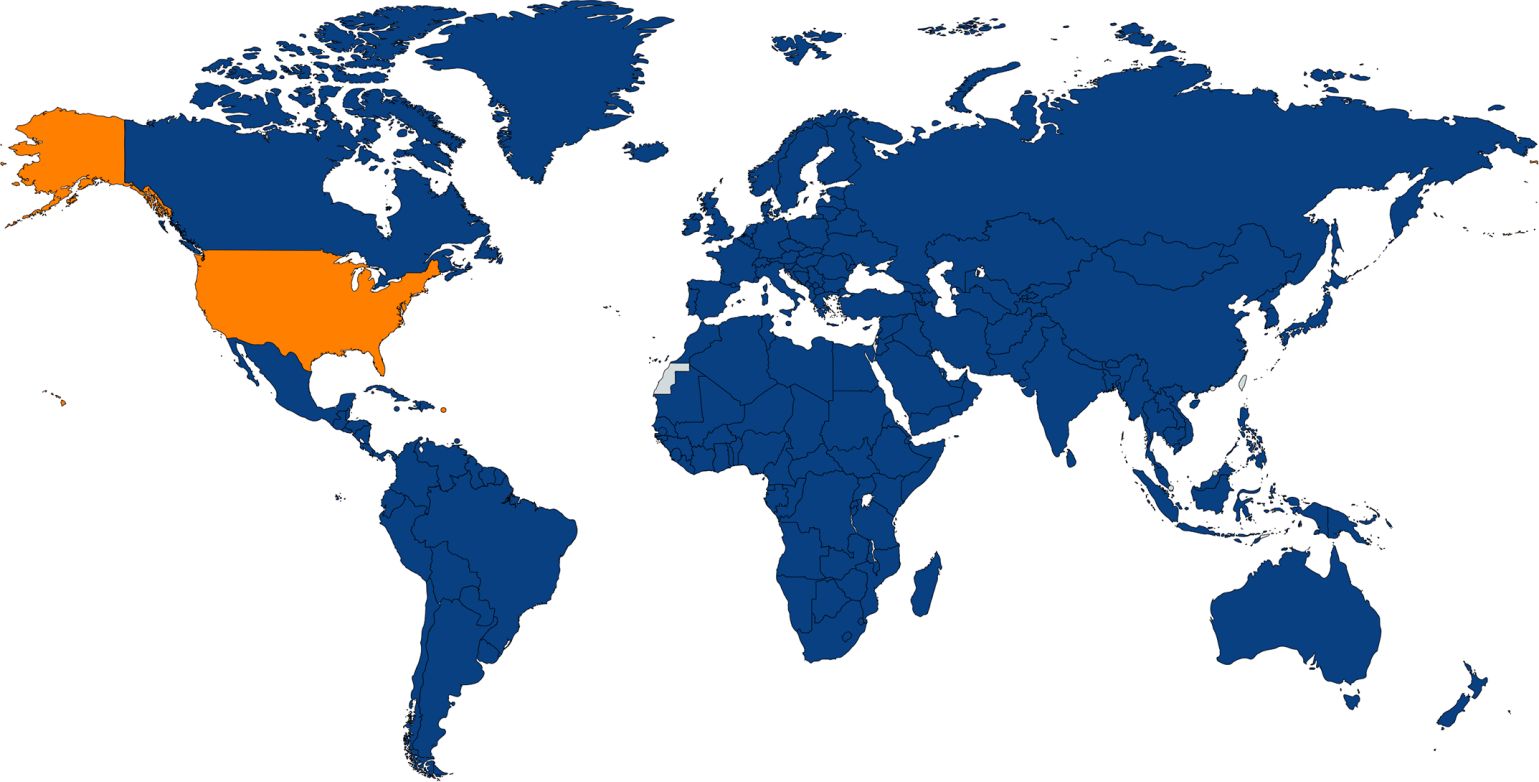
State parties to the CRC. Original figure created with Mapchart.net based on data from OHCHR (http://indicators.ohchr.org/). CRC, Convention on the Rights of the Child; OHCHR, Office of the UN High Commissioner for Human Rights.

Beyond failing to ratify a treaty, the rights of children in the US (as well as in Europe) are increasingly being violated due to a rise in intolerant ideologies and ignorance of basic scientific and medical facts. Here, we highlight several key examples in which we feel that children are being unnecessarily placed in harm’s way due to failure to heed established scientific evidence in lieu of ideological or personal beliefs (Box 1).

**Box 1. Science in the defense of children**

1. Promoting vaccine access
2. Alleviating child poverty
3. Treating and curing neglected diseases
4. Promoting resilience to climate change
5. Stopping gun violence

## Vaccines and the antivaccine movement

Tens of thousands of children in the US and in Europe are currently denied the right to be vaccinated against deadly pediatric infectious diseases such as measles, pertussis (whooping cough), and other conditions [3]. Because of misinformation about the safety of vaccines or about vaccines causing autism and other chronic conditions, parents are withholding or delaying life-saving vaccines from their children. Yet there is now overwhelming scientific evidence that there is no link between vaccines and autism [4]. There is also no plausibility for such a link based on the findings that the neurodevelopmental pathways of autism and related mental disabilities begin prenatally [4]. In spite of the science, vaccine coverage has declined precipitously in many communities, and as a result, there have been thousands of measles cases in Europe both last year and this year, including multiple measles deaths. In the US, there was a terrible measles outbreak in Minnesota in 2017, and in the most recent 2018 winter flu epidemic, most of the children who died had not been vaccinated [3]. Under the banner of terms such as “medical freedom” or “choice,” parents are, sadly, subjugating the rights of their children to be protected against disease out of misinformation and misplaced fear [3].

The latest information indicates that the consequences of vaccine exemptions have been dire, with 14,000 measles cases in Europe between April 2017 and March 2018, mostly in Italy, Romania, and Greece and mostly among those who had not been vaccinated [5]. More than 20,000 measles cases have occurred in Ukraine in 2018, with 11 deaths [6]. In the US, widespread vaccine exemptions for nonmedical, unwarranted reasons associated with philosophical or personal beliefs create a dangerous situation that could lead to European-style disease outbreaks [7].

## Child poverty, neglected diseases, immigration, and climate change

In 2018, Philip Alston, the UN Special Rapporteur on extreme poverty, delivered his report on America to the UN Human Rights Council, based on his fact-finding mission to the US the previous December [8]. The findings of the report were impressive for a number of reasons, including the depth and breadth of child poverty. For example, over 13 million American children live in poverty, with children constituting one-third of all people who live in poverty, especially black and Hispanic children [8]. Moreover, on any given night, more than 100,000 children are homeless, while more than 1 million students experience homelessness during the school year [8].

Child poverty is intimately connected with illness. Thus, the US infant mortality rate is approximately 50% higher than the Organization for Economic Cooperation and Development (OECD) average [8], and there is emerging evidence for widespread neglected diseases of poverty, including neglected tropical diseases such as toxocariasis and Chagas disease, especially in the US Gulf Coast states [9]. Yet another group at high risk for infectious and neglected diseases are the thousands of children living in detention centers on the US border with Mexico [10]. For these children, there is an urgent need to conduct active surveillance studies to determine the type and extent of diseases, as well as efforts to identify ways to prevent disease transmission. There is also an urgent need to conduct research and development in order to identify new technologies to combat these pediatric diseases of the vulnerable poor [9].

Promoting resilience to the devastation resulting from climate change and global warming must also become an essential component of protecting the health of children. According to UNICEF, the nutritional and health stress of a massive drought in East Africa now jeopardizes the lives of 16 million people [11]. In Ethiopia, about 8 million people are close to starvation, including more than 1 million children under age 5 [11].

## Pediatric gun violence

The statistics are largely self-explanatory. More than 25,000 American children were killed through gun violence between the years 1999 and 2016, including almost 200 infants, 200 one-year-olds, and almost 300 three-year-olds [12]. Today, firearm injuries are the third most common cause of death for America’s children [12], while more than 90% of all pediatric firearm deaths among children in wealthy nations now occur in the US, averaging about 4 deaths per day [12]. However, in 2018, the US National Institutes of Health provided a $5 million grant for a new Firearm-safety Among Children & Teens Consortium (FACTs) [13].

## Concluding comments

There is a growing abandonment of the human rights of children across America and in Europe. Through science, we may have an opportunity to combat this alarming trend. In the area of vaccines, the scientific community must raise its voice on the dangers that vaccine exemptions pose to children at risk for measles, influenza, human papilloma virus (HPV), and other childhood illnesses while refuting false claims that vaccines can cause autism or are unsafe. Similarly, child poverty places infants and children at high risk for emerging and neglected diseases, gun violence is killing on average four American children daily, and climate change is trapping a generation of children in a cycle of malnutrition and long-term health consequences. For each, there is a dearth of international, federal, state, and private support of research to seek innovative solutions and dissemination of findings for these and related issues. The scientific community must bring its enormous expertise to promoting the rights of children and protect them from poverty, illness, and violence. As part of this obligation, it should urge the US Congress to ratify the UNCRC.

## Abbreviations

FACTs: Firearm-safety Among Children & Teens Consortium
HPV: human papilloma virus
OECD: Organization for Economic Cooperation and Development
OHCHR: Office of the UN High Commissioner for Human Rights
UN: United Nations
UNCRC: UN Convention on the Rights of the Child
